# Thin-film fixed-bed reactor (TFFBR) for solar photocatalytic inactivation of aquaculture pathogen *Aeromonas hydrophila*

**DOI:** 10.1186/1471-2180-12-5

**Published:** 2012-01-13

**Authors:** Sadia J Khan, Robert H Reed, Mohammad G Rasul

**Affiliations:** 1 Centre for Plant and Water Sciences, Faculty of Sciences, Engineering and Health, CQUniversity, Rockhampton, QLD 4702, Australia

## Abstract

**Background:**

Outbreaks of infectious diseases by microbial pathogens can cause substantial losses of stock in aquaculture systems. There are several ways to eliminate these pathogens including the use of antibiotics, biocides and conventional disinfectants, but these leave undesirable chemical residues. Conversely, using sunlight for disinfection has the advantage of leaving no chemical residue and is particularly suited to countries with sunny climates. Titanium dioxide (TiO_2_) is a photocatalyst that increases the effectiveness of solar disinfection. In recent years, several different types of solar photocatalytic reactors coated with TiO_2 _have been developed for waste water and drinking water treatment. In this study a thin-film fixed-bed reactor (TFFBR), designed as a sloping flat plate reactor coated with P25 DEGUSSA TiO_2_, was used.

**Results:**

The level of inactivation of the aquaculture pathogen *Aeromonas hydrophila *ATCC 35654 was determined after travelling across the TFFBR under various natural sunlight conditions (300-1200 W m^-2^), at 3 different flow rates (4.8, 8.4 and 16.8 L h^-1^). Bacterial numbers were determined by conventional plate counting using selective agar media, cultured (i) under conventional aerobic conditions to detect healthy cells and (ii) under conditions designed to neutralise reactive oxygen species (agar medium supplemented with the peroxide scavenger sodium pyruvate at 0.05% w/v, incubated under anaerobic conditions), to detect both healthy and sub-lethally injured (oxygen-sensitive) cells. The results clearly demonstrate that high sunlight intensities (≥ 600 W m^-2^) and low flow rates (4.8 L h^-1^) provided optimum conditions for inactivation of *A. hydrophila *ATCC 3564, with greater overall inactivation and fewer sub-lethally injured cells than at low sunlight intensities or high flow rates. Low sunlight intensities resulted in reduced overall inactivation and greater sub-lethal injury at all flow rates.

**Conclusions:**

This is the first demonstration of the effectiveness of the TFFBR in the inactivation of *Aeromonas hydrophila *at high sunlight intensities, providing proof-of-concept for the application of solar photocatalysis in aquaculture systems.

## Background

Controlling infectious diseases is one of the main challenges faced by the fish farming industry [1]. A wide range of pathogenic microbes cause a variety of diseases, including furunculosis, infectious pancreatic necrosis, infectious salmon anaemia and amoebic gill disease, each of which results in extensive economic losses [2,3]. There is a growing awareness of the need to eliminate such pathogens by disinfecting the water in the aquaculture systems [4,5]. Disinfection is an effective treatment for many types of pathogenic microorganisms, including viruses, bacteria, fungi and protozoan parasites [6]. However, water disinfection remains a scientific and technical challenge [7]. The most commonly used techniques for water disinfection are chlorination, membrane filtration and ozone treatment [8] but antibiotics and biocides have also been used. Unfortunately all have disadvantages, particularly in relation to the generation of toxic by-products which may cause health risks to human consumers [9]. Additionally, some viral vaccines have been developed in the past two decades, but these are limited to selected viral pathogens and they are also extremely costly to produce and to administer [10].

Solar radiation is an alternative, low-cost, effective technology for water disinfection [11]. Solar disinfection normally refers to exposure of contaminated water to natural sunlight for a sufficient length of time to reduce the number of pathogenic microbes below the infective dose [5,12]. So far the most commonly employed method for solar disinfection is to expose contaminated drinking water kept in transparent plastic containers to full sunlight for at least 6 h [11,13] which is slow, and is not always feasible as a result of daily and seasonal variations in weather conditions.

Solar disinfection can be enhanced substantially by using certain photocatalysts such as the photoactive semiconductors TiO_2_, ZnO, Fe_2_O_3_, WO_3 _and CdSe. These photocatalysts produce highly reactive oxygen species (ROS) which destroy microbial pathogens; this is known as solar photocatalytic disinfection [14,15]. Titanium dioxide (TiO_2_) is one of the most widely used, stable and active photocatalysts in water disinfection [8]. It has shown its effectiveness not only in small-scale solar disinfection reactors but also in pilot studies of large-scale solar photocatalysis for drinking water and waste water [16-19].

Typically, TiO_2 _slurries are used for chemical and microbial photodegradation [9,19]. However, such slurries create problems in separating the photocatalyst from the treated water, leading to the development of reactors containing an immobilised photocatalyst. Different types of solar photocatalytic reactors have been developed for water treatment [20]. The most frequently used types of reactors are: (i) the parabolic trough reactor (PTR), (ii) the double skin sheet reactor (DSSR), (iii) the compound parabolic collecting reactor (CPCR) and (iv) the thin-film fixed-bed reactor (TFFBR). The most important part of a TFFBR is a sloping plate coated with P25 TiO_2 _DEGUSSA over which flows the contaminated water during use. The TFFBR also contains a pump, by which the water flow rate can be controlled. The main advantages of this TFFBR are (i) its high optical efficiency, (ii) it's simple construction method and (iii) the low investment costs involved in development. Further advantages are that oxygen transfers effectively into the water film and there is no need for TiO_2 _separation from the treated water, in contrast to reactors based on TiO_2 _slurries.

An understanding of the mechanism of microbial photoinactivation during solar photocatalysis comes mostly from studies of bacteria [5,7,21]. The most common photocatalytic inactivation mechanism described is based on inactivation due to hydroxyl radicals and other reactive oxygen species (ROS) when bacteria come in contact with a solar-excited photosensitiser. This photooxidation process causes cell membrane disruption and increase cellular permeability, with significant cell damage that eventually results in complete inactivation of the bacteria [13].

The conventional approach to assessing the viability of bacteria during solar disinfection is to enumerate samples after exposure to sunlight, using conventional plate counts on a suitable agar-based growth medium with incubation of plates in standard aerobic conditions (e.g. 24 h incubation at a suitable temperature). However, recent studies have demonstrated that reactive oxygen species (ROS), derived mainly from aerobic respiration during the enumeration process, may inactivate sub-lethally damaged bacteria and prevent their growth and enumeration under aerobic conditions [22]. Such injured cells can only be cultured and counted under conditions where reactive oxygen species are neutralised (ROS-neutralised conditions) e.g. by supplementing the growth medium with the peroxide scavenger sodium pyruvate and incubating under anaerobic conditions to prevent cellular respiration, allowing the bacteria to grow by fermentation [22-24]. This approach was taken in the present study; uninjured bacteria were enumerated under aerobic conditions while uninjured plus injured (ROS-sensitive) bacteria were enumerated under ROS-neutralised conditions, with the difference between the counts under both sets of conditions representing the number of injured bacteria in the sample.

Even though bacteria have received more attention than other groups of microbes in solar photocatalysis research, bacterial pathogens of fish have been largely ignored in these studies, prompting the study reported here. *Aeromonas hydrophila *is a Gram-negative bacterium, known to be a primary fish pathogen [25]. *A. hydrophila *tends to be virulent towards most cultured and wild freshwater fish, especially trout, salmon, carp, catfish and tilapia. Red fin diseases and haemorrhagic septicaemia are mainly associated with *A. hydrophila *[26]. Antibiotics and several vaccines have been used to treat these infections, but extensive use of antibacterial agents has caused *A.hydrophila *to develop resistance towards certain antibiotics including, ampicillin, tetracycline, chloramphenicol and sulphonamides, [27]. Consequently, it is now important to develop alternative treatments for this pathogen.

The present research reports on the development of a system for the disinfection of water contaminated with *A. hydrophila *ATCC 35654 as a model for solar photocatalysis in aquaculture systems. The result presented here show for the first time that solar photocatalysis can provide an effective means of inactivation of *A.hydrophila*, which provides proof-of-concept for the application of solar photocatalysis in aquaculture systems.

## Methods

### Reactor

**Figure 1:**
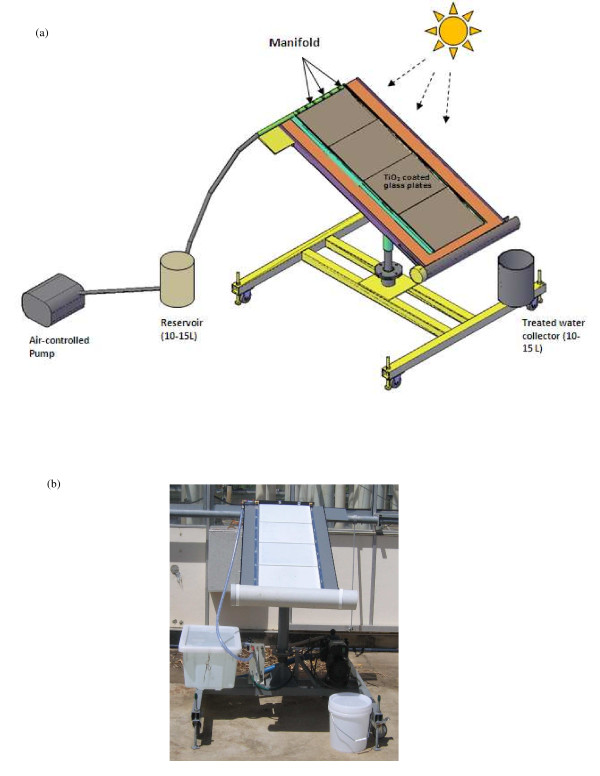
**(a) schematic diagram and (b) photograph of the thin-film fixed-bed reactor (TFFBR) used in this study**.

A pilot-scale thin-film fixed-bed reactor (TFFBR) system has been developed, based on two previous researches [28,29]. The overall experiment was set-up as a single-pass process and the reactor consisted of a water reservoir (representing an aquaculture pond in the model system), an air-controlled pump, a solar collector (glass plate) with immobilised photocatalyst, P25 TiO_2 _DEGUSSA and a collector vessel for the treated water (Figure 1). As in previous studies of chemical degradation [28,29] and recent studies of microbial inactivation [7,21], the reactor angle was maintained at 20° throughout, and the light intensity was measured from the same angle as that of the reactor. The illuminated surface area was 1.17 m in depth and 0.40 m in width; the irradiated volume was 200 mL in 2.5 min (irradiance time) and the density of the TiO_2 _photocatalyst 20.50 g m^-2 ^and the photocatalyst layer was not covered during the experiments.

The TiO_2 _P25 Degussa photocatalyst was coated on four pieces of 3.3 mm thick Borofloat 33 glass plates (Schott, Australia). Plates were degreased using a reagent grade Piranha solution (3:1 sulphuric acid and 30% hydrogen peroxide). Then a slurry of TiO_2 _was prepared with methanol and the glass was coated by spraying. Then it was baked at 450°C for 2 h to anneal the TiO_2 _to the glass.

### Bacterial culture

*Aeromonas hydrophila *ATCC 35654 was purchased from Oxoid, Australia. This was maintained by repeated sub-culture on trypticase soy agar (TSA) (Oxoid, Australia) at 25°C. The stock cultures were stored at-70°C in sterile saline containing 20% (v/v) glycerol. For experimental use, cultures were prepared by loop inoculation of bacteria into 100 mL of trypticase soy broth (TSB) (Oxoid, Australia) on a shaking water bath for 24 h at 25°C. To obtain a working cell suspension, the overnight culture was centrifuged at 13000 g for 1 min. The supernatant was discarded and the cell pellet was rinsed twice with water prepared by reverse osmosis, to remove all traces of the growth medium. Then 6 mL of this cell suspension was added to the 6 L of sterile natural spring water (Satur8 Pty. Ltd, Australia) to give an initial bacterial count of 10^5 ^CFU/ml added to the reservoir of the reactor.

### Experimental procedure

For each experiment water containing *A.hydrophila *ATCC 35654 was run from the reservoir through the reactor for at least 30 min with different flow rates (4.8 L h^-1^, 8.4 L h^-1^and 16.8 L h^-1^) controlled by an air-pressure pump. Every 10 min a water sample was collected in a sterile McCartney bottle from the outflow of the TiO_2_-coated plate, labelled and returned to the laboratory, shielded from further exposure to sunlight. Reservoir samples were also collected at 0 min and 30 min to provide the untreated (dark) control counts for each experiment. During the experiment, every 2 min, total sunlight intensity readings were obtained in W/m^2 ^using a Pyranometer (model SP1110, Skye instruments, UK). At the same time solar ultra-violet (UV) light intensity readings were also measured using a Solarmeter (model 5.0, UV meters, Solartech, Inc, USA). Experiments were carried out under different sunlight conditions with a range of total sunlight of 300-1200 W m^-2 ^and UV intensities of 20-60 W m^-2^. A comparative experiment was also carried under full sunlight (> 1000 W m^-2^) with the same procedure using a glass plate of the same size but without TiO_2 _in the TFFBR at 4.8 L h^-1^.

### Laboratory enumeration

Each sample was processed by serial decimal dilution to cover the range 10^0^-10^-2^. Then three aliquots of 20 μL of each dilution were plated by the droplet spread technique [23] on TSA with or without 0.05% w/v sodium pyruvate and incubated at 25°C for 48 h. Plates without sodium pyruvate were incubated in a conventional aerobic incubator (Cotherm, Biocell 1000, Thermo Fisher Scientific Ltd. Australia), to provide counts of healthy bacteria. Plates with sodium pyruvate were incubated under anaerobic condition in a dedicated anaerobic cabinet (Model 10, COY Inc., USA) to create ROS-neutralised conditions, giving the count of healthy bacteria plus injured bacteria. Plates were counted using a colony counter and converted to log_10 _CFU/mL. To provide a measure of the inactivation that occurred due to solar photocatalysis, the log-transformed count of sunlight-treated water at each time point were subtracted from the log-transformed count of untreated water (dark control) to give an overall value for log inactivation. As an example, for a treated log count of 3.83 and an untreated log count of 5.16, then log inactivation = 5.16-3.83 = 1.33, which represents (antilog 1.33) a reduction in absolute count of around twenty-fold.

Statistical comparisons of different data sets were carried out using regression analysis of log-transformed data.

## Results

### Effectiveness of TiO_2 _photocatalyst on inactivation of *A. hydrophila *inactivation

**Figure 2:**
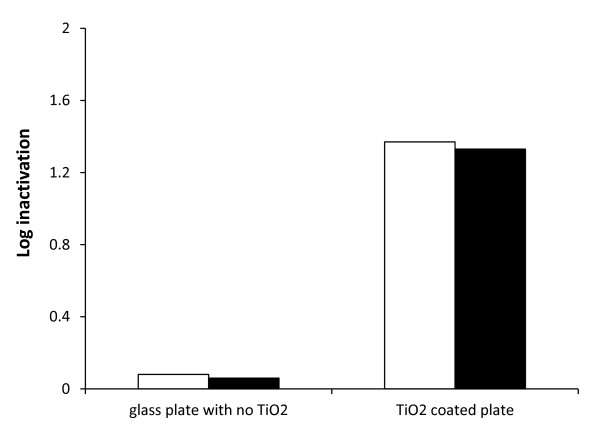
**Effect of TiO_2 _photocatalyst on inactivationof *A. hydrophila *(ATCC 35654) under high sunlight condition (1032-1187) W m^-2 ^or (UV light intensity = 50.8-62.8 W m^-2^) at 4.8 L h^-1^, with and without TIO_2 _coating on the TFFBR single pass reactor**. Enumeration was carried out under standard aerobic conditions (unfilled bars) and under ROS-neutralised condition (filled bars).

In Figure 2, spring water with an initial level of 5.16 Log CFU ml^-1 ^*Aeromonas hydrophila *(ATCC 35654) showed only 0.06 log inactivation with a single pass across the glass plate reactor (no TiO_2_) with a final average concentration of 5.1 log CFU ml^-1 ^and with no detectable cell injury, under high sunlight intensity of (1032-1187) W m^-2 ^(UV light intensity = 52.8-62.8 W m^-2^). On the other hand, a single pass across the TFFBR with TiO_2 _showed 1.33 log inactivation, with minimal cell injury, with an average final concentration of 3.83 Log CFU ml^-1 ^from a similar 5.16 Log CFU ml^-1^, initial level of *A. hydrophila*.

### Interrelationship of flow rate and total sunlight on inactivation of *Aeromonas hydrophila*

**Figure 3:**
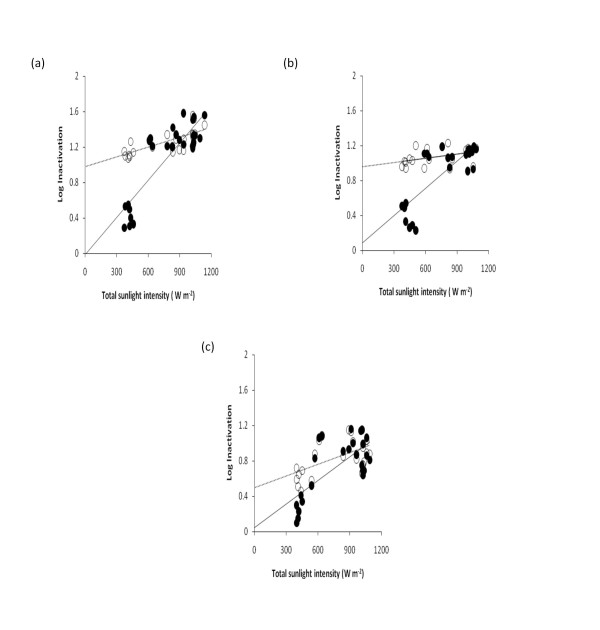
**Effect of different flow rates (a) 4.8 L h^-1^, (b) 8.4 L h^-1 ^and (c) 16.8 L h^-1^, on log inactivation of *A.hydrophila *ATCC 35654 in spring water run through the TFFBR under different total sunlight conditions**. Enumeration was aimed at under standard aerobic conditions (open circle) and under ROS-neutralised conditions (closed circle).

Figure 3a shows the log inactivation data for *A.hydrophila *ATCC 35654 in sterile spring water run through the TFFBR at 4.8 L h^-1 ^flow rate under various total sunlight conditions, from 300 W m^-2 ^to 1200 W m^-2^, and then enumerated under (i) aerobic and (ii) ROS-neutralised conditions. Thus, each experiment provides two sets of log inactivation data, (i) an aerobic result, based on healthy cells only and (ii) a ROS-neutralised result, representing healthy and injured cells together. At low total sunlight intensities of < 600 W m^-2^, there was a far larger difference between the log-inactivation values obtained using aerobic and ROS-neutralised counts than was the case for sunlight intensities above 600 W m^-2^. This demonstrates a far greater proportion of injured (ROS-sensitive) cells at lower sunlight conditions (< 600 W m^-2^). In contrast, higher sunlight intensities ranging from 600 W m^-2 ^to 1100 W m^-2 ^resulted in greater proportional inactivation (higher log inactivation values), whether quantified both in aerobic or ROS-neutralised conditions, with minimal differences in log inactivation values. This demonstrates that at high sunlight intensities, inactivation is not accompanied by sub-lethal injury, in contrast to the findings at lower sunlight intensities (< 600 W m^-2^).

Linear regression trend lines were plotted for each data set (i.e. for log inactivation data obtained from counts under aerobic and ROS-neutralised conditions). ROS-neutralised condition predicted a best fit line with an intercept close to zero and a strong fit of the data to the trend line, based on a regression coefficient of 0.751 (Table 1). In contrast under aerobic conditions, the trend line has a positive intercept and a weaker fit, with a regression coefficient of 0.535. Given that the logical expectation is that there would be no inactivation at 0 W m^-2 ^sunlight, this is consistent with the notion that data based on ROS-neutralised counts provide a more appropriate measure of inactivation than standard aerobic counts, with the latter give a substantial overestimate of the effectiveness of solar photocatalysis at low sunlight intensities (< 600 W m^-2^).

**Table 1:** Linear regression analysis for inactivation of *A.hydrophila *ATCC 35654 under 3 different flow rates

Flow rate	Enumeration condition	Linear regression equation	R^2 ^values
4.8 L h^-1^	Aerobic	Y = 0.0004X+0.976	0.535
	
	ROS-neutralised	Y = 0.0018X-0.010	0.751

8.4 h^-1^	Aerobic	Y = 0.0002X+0.981	0.179
	
	ROS-neutralised	Y = 0.0012X+0.084	0.650

16.8 L h^-1^	Aerobic	Y = 0.0004X+0.496	0.311
	
	ROS-neutralised	Y = 0.0009X+0.048	0.503

Figure 3b and 3c showed the log inactivation data for *A.hydrophila *ATCC 35654 in spring water run through the reactor at flow rates of 8.4 L h^-1 ^and 16.8 L h^-1^, respectively, under equivalent sunlight conditions to those shown in Figure 3a. Both graphs show a similar pattern of greater proportional cell injury, manifest as ROS-sensitivity and lack of growth under aerobic conditions, to the data for low flow rate (Figure 3a) when the total sunlight intensity was < 600 W m^-2^. Similarly, when the total sunlight intensity was 600-1100 W m^-2^, there was a greater log inactivation and less evidence of sub-lethal injury.

Linear regression analyses were also carried out for flow rate data at 8.4 and 16.8 L h^-1^. At both flow rates, the trend lines based on aerobic counts gave positive intercepts whereas the ROS-neutralised data showed an intercept close to zero, in line with the outcome at 4.8 L h^-1 ^(Table 1). Similarly, the aerobic count data at 8.4 and 16.8 L h^-1 ^had lower regression coefficients than for ROS-neutralised data. Overall, the interpretation of these data is that aerobic counts overestimate the apparent inactivation of *A. hydrophila *ATCC35654 and that ROS-neutralised counts are required to provide counts of injured and healthy cells, with trend lines that fit with the logic of a zero intercept and a strong fit of the data to the trend line. Based on ROS-neutralised data, there is a strong effect of flow rate on photocatalysis using the TFFBR--this is evident from the decrease in slope for the linear regression analysis based on the ROS-neutralised data from the slowest flow rate (4.8 L h^-1^) to the fastest flow rate (16.8 L h^-1^), shown in Table 1. An equivalent change was not observed for aerobic data, which again points to the issues around low aerobic counts at low sunlight intensities and their effects on the overall trend data.

The data in Figure 3 also demonstrate that the combination of a low flow rate of 4.8 L h^-1 ^combined with a total sunlight intensity of 600 W m^-2 ^or more gave the greatest log inactivation of *A. hydrophila *ATCC 35654, pointing to such conditions as being most effective for solar photocatalysis.

### Interrelationship of flow rate and solar UV on inactivation of *Aeromonas hydrophila*

**Figure 4:**
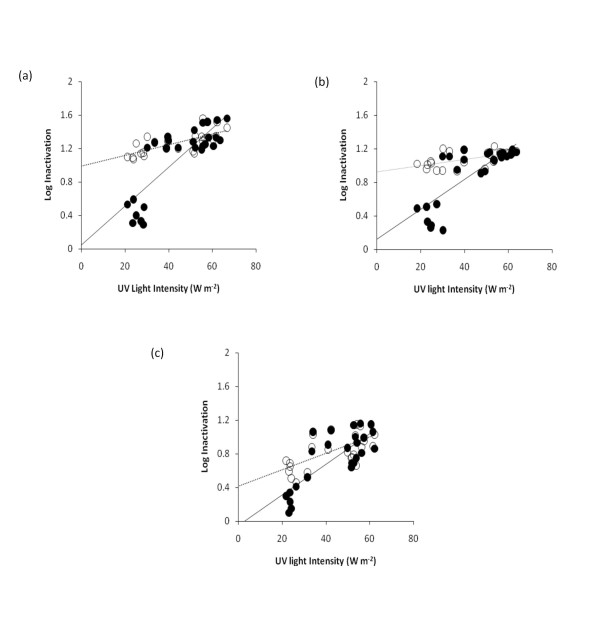
**Effect of different flow rates (a) 4.8 L h^-1^, (b) 8.4 L h^-1 ^and (c) 16.8 L h^-1^, on log inactivation of *A.hydrophila *ATCC 35654 in spring water run through the TFFBR under different Ultraviolet (UV) light conditions**. Enumeration was aimed at under standard aerobic condition (open circle) and under ROS-neutralised condition (closed circle).

Figure 4 shows the log inactivation rate of *A.hydrophila *(ATCC 35654) in spring water run through the reactor with 3 flow rates (4.8, 8.4 and 16.8 L h^-1^), with the data plotted against solar UV intensity, ranging from 20 W m^-2 ^to 80 W m^-2^, to see whether the same results were obtained as for total sunlight in Figure 3. This was carried out because TiO_2 _is specifically photoactivated by UV light at 390-400 nm. Overall, the same trends of (i) positive intercepts for log inactivation data based on aerobic counts (ii) close-to-zero intercepts for log inactivation data based on ROS-neutralised counts (Table 2) and (iii) weaker fits of trend lines based on aerobic counts were observed for results plotted against UV light as those for total sunlight (Figure 3), with no evidence of any stronger relationships based on UV data than those for total sunlight. This demonstrates that total sunlight is as good a predictor of solar photocatalysis in these TFFBR experiments as UV light.

**Table 2:** Linear regression equations and R^2 ^values of *A.hydrophila *ATCC 35654 inactivation against UV light intensities under 3 different flow rates

Flow rates	Enumeration condition	Linear regression equation	R^2 ^values
4.8 L h^-1^	Aerobic	Y = 0.0006X+0.985	0.492
	
	ROS-neutralised	Y = 0.023X+0.050	0.678

8.4 L h^-1^	Aerobic	Y = 0.004X+0.961	0.410
	
	ROS-neutralised	Y = 0.018X+0.120	0.639

16.8 L h^-1^	Aerobic	Y = 0.009X+0.415	0.395
	
	ROS-neutralised	Y = 0.018X-0.052	0.611

## Discussion

While earlier studies have mostly concentrated on the application of TFFBR systems for chemical degradation, TiO_2_-based photocatalysis has proved its ability to enhance the rate of inactivation of microbes in contaminated drinking waters and waste waters, enabling such waters to be disinfected [20,21]. The present study has clearly shown that *A. hydrophila *ATCC 35654 can be effectively inactivated in spring water using the TFFBR under sunlight conditions of > 600 W m^-2^, demonstrating its potential for applications in aquaculture, especially in tropical and sub-tropical developing countries where sunlight is abundant and the resources for alternative forms of disinfection are scarce.

The efficiency of the TFFBR was also investigated in this study by flowing (at 4.8 L h^-1^) contaminated spring water sample under high sunlight intensities and by using same sized glass with and without TiO_2 _under the same reactor conditions. The findings of this study confirm the results of two previous studies [7,21]. The presence of TiO_2 _showed a clear enhancement in solar photocatalysis [21]. The current study clearly shows that solar energy alone is unsufficient to inactivate *A. hydrophila *and that a photocatalyst such as, TiO2 is required for effective reduction in counts.

Microbial disinfection by solar photocatalysis is a complex and challenging process [30]. The extent of inactivation observed in *A. hydrophila *ATCC 35654 under high sunlight intensity was also found to be similar to that reported for other microbes in early studies [8,16]. Thus one investigation showed that when the UV irradiance was 20-43 W m^-2^, the inactivation of the fungus *Fusarium *sp. was faster than than at lower irradiances (cloudy weather condition), using a CPC reactor [8]. Similar effects of solar irradiation on inactivation were observed in the present study, under different sunlight condition. For example, at lower sunlight conditions (total sunlight intensity = 300-600 W m^-2 ^or UV irradiance = 20-40 W m^-2^) inactivation was considerably less than was observed at the highest sunlight conditions (> 1100 W m^-2 ^and > 65 W m^-2^) at 4.8 L h^-1^. Solar photocatalytic activity was also demonstrated for various pathogens in drinking water in a batch culture reactor using simulated sunlight [16], in contrast to the TFFBR system tested under natural sunlight used in the present study. Similarly, recent studies have succeeded in photocatalysis but they required a long UV exposure times to achieve a log inactivation of 6-fold for *E.coli *K12 using a CPC pilot plant solar reactor [7,21]. Such inactivation is far greater than that observed in the present study, where the log inactivation was around 1.38 with an average initial count of 1.36 × 10^5 ^CFU mL^-1 ^and average final count of 5.10 × 10^3 ^CFU mL^-1^, at the highest sunlight intensities--this is most likely due to the rapid transfer of contaminated liquid across the TFFBR plate, which is around 2.5 min at 4.8 L h^-1^flow rate, in the present study. As most previous studies have used an artificial UV light source for exposure, it is difficult to make direct comparisons to the present study, where natural sunlight has been used. Additionally, different type of reactors will have different dynamics of inactivation and flow, as well as dissimilar kinetics of change with light intensity.

Counts of *A. hydrophila *ATCC 35654 exposed to the TFFBR system at low sunlight (< 600 W m^-2^) under ROS-neutralised conditions were substantially higher than those obtained from standard aerobic plate counts, which validates the finding from previous studies of *E. coli *and other bacteria [22-24]. This indicates that the antioxidant system of many cells of *A. hydrophila *ATCC 35654 was damaged by solar photocatalysis at low sunlight intensities, resulting in their sensitivity towards their own respiratory by-products. Such cells were only able to form colonies when sodium pyruvate (a scavenger of hydrogen peroxide) is added, coupled with growth under anaerobic conditions, which will enable the bacteria to use fermentative pathways, rather than aerobic respiration, for energy generation. The findings of this present study unequivocally demonstrate that at all three different flow rates tested, at low sunlight intensities (< 600 W m^-2^) there was a substantial difference between the log inactivation results based on ROS-neutralised and conventional aerobic counts (Figures 3 and 4). At 4.8 L h^-1^, there was close to 1 log difference between the ROS-neutralised and aerobic log inactivation results, suggesting that the aerobic data provide an apparent inactivation that overestimates the true value. For other two flow rates (8.4 and 16.8 L h^-1^) the difference between the two sets of data were around 0.9 and 0.5 (with similar initial inoculam of 1.33 × 10^5 ^CFU mL ^-1 ^and final count of 9.40 × 10^3 ^and 1.75 × 10^4 ^CFU mL^-1^) respectively, indicating a reduction in the amount of sub-lethal injury at higher flow rates that is also coupled with a lower overall inactivation (Table 1). While previous studies of solar disinfection have demonstrated sub-lethal injury and ROS-sensitivity in batch culture with uncalatysed reactors, this is the first study to do so for the TFFBR continuous flow photocatalytic system. On the other hand, at higher sunlight intensities (> 600 W m^-2^), the differences between the results based on aerobic counts and ROS-neutralised counts were negligible for all flow rate conditions, demonstrating the strength of high sunlight to provide powerful inactivation, with no sign of sub-lethal injury.

Sometimes, sunlight itself is not sufficient for water disinfection, due to the effectiveness of photoreactivation mechanisms in microorganisms [31]. A recent study has demonstrated the effectiveness of immobilised TiO_2 _reactors in inactivating bacteria to such an extent that their photoreactivation mechanisms are not able to repair the damage [19], indicating that fixed-bed TiO_2 _reactors increase the extent of damage to bacteria from the very beginning of the process, whereas TiO_2 _slurry systems required longer irradiation times to cause an equivalent amount of cellular damage. In a slurry system, TiO_2_-related damage occurs at the cell membrane of bacteria; however, damage is distributed across the whole membrane, so membrane permeability effects are not always strong enough to cause irreversible inactivation in the early stages of the process. On the other hand, in a fixed-bed reactor, while the free radicals generated may be lower in number, the damage can be concentrated on the cell membrane area, causing inactivation [19]. The result of the current study can be interpreted in similar approach, but with respect to sunlight intensity. Here it was observed that while low sunlight resulted in substantial sub-lethal injury, with results based on ROS-neutralised counts being far lower than for aerobic data, at higher light intensities, ROS neutralised data were similar to those based on aerobic counts. As the data at high sunlight intensities showed little evidence of sub-lethal injury, this demonstrates that the TFFBR system will be more efficient in full sunlight, where maximum inactivation is achieved.

The dynamics of flow rate in pilot-scale photocatalytic reactors have not been well studied to date. In considering treating large volumes of water, as in aquaculture systems, it is obvious that flow rate will be a crucial parameter. A pilot-scale CPC reactor using TiO_2 _in suspension with different flow rates has been used to study the inactivation of *Fusurium *sp. spores [18]; achieving the highest inactivation rate of *Fusurium *spores at a flow rate of 30.0 L min^-1 ^with added TiO_2 _at 100 mg L^-1 ^concentration. However, such systems require separation of the suspended TiO_2 _after treatment, which adds to the complexity, in contrast to immobilised systems such as the TFFBR. Another recent solar disinfection study also showed the importance of evaluating different parameters including: flow rate; water volume within the reactor; temperature; and solar energy [32]. They used a CPC reactor with no added TiO_2 _and suggested that increasing flow rate has a substantial negative effect on the inactivation of bacteria, which is in agreement with the flow rate investigations of the present study. Here, the lowest flow rate of 4.8 L h^-1 ^was found to be the most effective for inactivation of *A. hydrophila *ATCC 35654 as the residence time of 2.5 minin the 4.8 L h^-1 ^experiment is almost twice as high as the 8.4 L h^-2 ^experiment.(86 s) Similarly, when the total sunlight intensity is at average of 1000 W m^-2^, the cumulative energy, 150 KJ m^-2 ^at 4.8 L h^-1 ^is higher than that of 86 KJ m^-2 ^at 8.4 L h^-1 ^which will play a major role *A. hydrophila *inactivation. In this study, the water temperature in the reservoir was maintained at (22-23)°C throughout the experiments. Due to the open structure of the TFFBR, the temperature of the water on the reactor plate was not measured, though it is logical to expect that it would be positively related to sunlight intensity.

## Conclusion

The results clearly demonstrate that high sunlight intensities (> 600 W m^-2^) and low flow rates (4.8 L h^-1^) provide optimum conditions for the inactivation of the fish pathogen *A. hydrophila *ATCC 35653, with fewer injured (ROS-sensitive) cells under such conditions than at lower sunlight intensities. Using a TFFBR system to disinfect these bacteria under natural sunlight is a novel and alternative approach to conventional chemical disinfectants and antibiotics for control of this pathogen. The present study is also the first to report sub-lethal injury for a solar photocatalytic system at low sunlight intensities (< 600 W m^-2^), which places a question mark over conventional aerobic counts under such conditions and demonstrates that ROS-neutralised conditions are required to enumerate survivors of solar photocatalysis at low sunlight levels. However, conventional aerobic counts should be effective in enumerating *A. hydrophila *ATCC 35653 surviving a TFFBR system operating under high sunlight conditions, making it easier to assess efficiency under such conditions. Overall, the use of solar photocatalysis represents a potential low-cost, sustainable approach across all countries with consistent sunny climates.

## Competing interests

The authors declare that they have no competing interests.

## Authors' contributions

The project was designed by SK, RR and MR. All experiments were performed by SK under supervision of RR. The paper was co-drafted by SK and RR. All authors approved the final version of the manuscript.

## References

[B1] Eiras JC Segner H Wahil T Kapoor BG Fish diseases 2008 Science publishers

[B2] Murray AG Peeler EJ A framework for understanding the potential for emerging diseases in aquaculture Prev Vet Med 2005 67 223 235 10.1016/j.prevetmed.2004.10.012 15737433

[B3] Pulkkinen K Saumalainen LR Read AF Ebert P Rinimaki P Vatonen ET Intensive fish farming and the evolution of pathogen virelence: the case of Columnaris disease in Finland Proceedings of Royal society B 2010 277 593 600 10.1098/rspb.2009.1659 PMC2842694 19864284

[B4] Sharrer MJ Summerfelt ST Ozonation followed by ultraviolet irradiation provides effective bacteria inactivation in a freshwater recirculating system Aquacult Eng 2007 37 2 180 191 10.1016/j.aquaeng.2007.05.001

[B5] Berecz MJ The disinfection and protection of microorganism in complex water systems' PhD thesis 2010 University of North Carolina, Biomedical science department

[B6] Gamage J Zhang Z Applications of Photocatalytic Disinfection Int J Photoenergy 2010 Article ID 764870. doi:10.1155/2010/764870

[B7] Van Grieken R Marugán J Pablos C Furones L López A Comparison between the photocatalytic inactivation of Gram-positive *E. faecalis *and Gram-negative *E. coli *faecal contamination indicator microorganisms Appl Catal B Env 2010 100 1-2 212 220 10.1016/j.apcatb.2010.07.034

[B8] Sichel C De Cara M Tello J Fernández-Ibáñez P Effect of UV solar intensity and dose on the photocatalytic disinfection of bacteria and fungi Catal Today 2007 129 152 160 10.1016/j.cattod.2007.06.061

[B9] Blanco-Galvez J Fernandez-Ibanez P Malato-Rodriguez S Solar photocatalytic detoxification and disinfection of water: recent overview J Sol Energ Engineering 2007 129 1 4 15 10.1115/1.2390948

[B10] Lorenzen N LaPatra SE DNA vaccines for aquacultured fish Rev Sci Tech Off Int Epiz 2005 24 1 201 213 16110889

[B11] Byrne JA Fernandez-Iba˜nez PA Dunlop PSM Alrousan DMA Hamilton JJ Photocatalytic enhancement for solar disinfection of water: a review Int J Photoenergy 2011 Article ID 798051, doi:10.1155

[B12] Ubomba-Jaswa E Fernández-Ibáñez P Navntoft C Polo-López MI McGuigan KG Investigating the microbial inactivation efficiency of a 25 L batch solar disinfection (SODIS) reactor enhanced with a compound parabolic collector (CPC) for household use J Chem Tech Biotechnol 2010 85 8 1028 1037 10.1002/jctb.2398

[B13] Alrousan DMA Dunlop PSM McMurray TA Byrne JA Photocatalytic inactivation of *E. coli *in surface water using immobilised nanoparticle TiO_2 _films Water Res 2009 43 1 47 54 10.1016/j.watres.2008.10.015 19007965

[B14] Reed RH The inactivation of microbes by sunlight; solar disinfection as a water treatment process Adv Appl Microbiol 2004 54 333 356 15251286 10.1016/S0065-2164(04)54012-1

[B15] McCullagh C Robertson J Bahnemann D Robertson P The application of TiO_2 _photocatalysis for disinfection of water contaminated with pathogenic micro-organisms: a review Res Chem Intermediat 2007 33 3 359 375 10.1163/156856707779238775

[B16] Lonnen J Kilvington S Kehoe SC Al-Touati F McGuigan KG Solar and photocatalytic disinfection of protozoan, fungal and bacterial microbes in drinking water Water Res 2005 39 5 877 883 10.1016/j.watres.2004.11.023 15743634

[B17] Maneerat C Hayata Y Antifungal activity of TiO_2 _photocatalysis against *Penicillium expansum *invitro and in fruit tests Int J Food Microbiol 2006 107 2 99 103 10.1016/j.ijfoodmicro.2005.08.018 16269195

[B18] Polo-López MI Fernández-Ibáñez P García-Fernández I Oller I Salgado-Tránsito I Sichel C Resistance of *Fusarium *sp spores to solar TiO2 photocatalysis: influence of spore type and water(scaling up results) J Chem Tech Biotech 2010 85 8 1038 1048 10.1002/jctb.2397

[B19] Pablos C van Grieken R Marugán J Moreno B Photocatalytic inactivation of bacteria in a fixed-bed reactor: mechanistic insights by epifluorescence microscopy Catal Today 2011 161 1 133 139 10.1016/j.cattod.2010.10.051

[B20] Malato S Fernández-Ibáñez P Maldonado MI Blanco J Gernjak W Decontamination and disinfection of water by solar photocatalysis: recent overview and trends Catal Today 2009 147 1 1 59 10.1016/j.cattod.2009.06.018

[B21] Sordo C Van Grieken R Marugán J Fernández-Ibáñez P Solar photocatalytic disinfection with immobilised TiO_2 _at pilot-plant scale Water Sci Technol 2010 61 2 507 512 10.2166/wst.2010.876 20107278

[B22] Khaengraeng R Reed RH Oxygen and photoinactivation of *Escherichia coli *in UVA and sunlight J Appl Microbiol 2005 99 39 50 10.1111/j.1365-2672.2005.02606.x 15960663

[B23] Tandon P Chhibber S Reed HR Inactivation of *Escherichia coli *and coliform bacteria in traditional brass and earthernware water storage vessels Anton Van Lee 2005 88 1 35 48 10.1007/s10482-004-7366-6 15928975

[B24] Sharan R Chhibber S Attri S Reed R Inactivation and injury of *Escherichia coli *in a copper water storage vessel: effects of temperature and pH Anton Van Lee 2010 97 1 91 97 10.1007/s10482-009-9395-7 19924559

[B25] Austin B Austin A Bacterial fish pathogens: disease of farmed and wild fish 1999 3 Springer and Praxis publications

[B26] LaParta SE Plant KP Alcorn S Ostland V Winton J An experimental vaccine against *Aeromonas hydrophila *can induce protection in rainbow trout, Oncorhynchus mykiss (Walbaum) J Fish Dis 2010 33 143 151 10.1111/j.1365-2761.2009.01098.x 19732266

[B27] Woo PTK Bruno DW Fish diseases and disorders 3 1999 Wallingford: CABI publishing

[B28] Bekbölet M Phtocatalytic bacterocidal activity of TiO_2 _in aqueous suspensions of *E. coli* Water Sci Technol 1997 35 95 100

[B29] Bahnemann D Photocatalytic water treatment: solar energy applications Solar Energy 2004 77 5 445 459 10.1016/j.solener.2004.03.031

[B30] Marugán J van Grieken R Pablos C Sordo C Analogies and differences between photocatalytic oxidation of chemicals and photocatalytic inactivation of microorganisms Water Res 2010 44 3 789 796 10.1016/j.watres.2009.10.022 19906399

[B31] Herrera Melián JA Doña Rodríguez JM Viera Suárez A Tello Rendón E Valdés do Campo C Arana J Pérez Peña J The photocatalytic disinfection of urban waste waters Chemosphere 2000 41 3 323 327 10.1016/S0045-6535(99)00502-0 11057593

[B32] Ubomba-Jaswa E Navntoft C Polo-Lopez MI Fernandez-Ibanez P McGuigan KG Solar disinfection of drinking water (SODIS): an investigation of the effect of UV-A dose on inactivation efficiency Photoch Photobio Sci 2009 8 5 587 595 10.1039/b816593a 19424529

